# A new movie is about to premiere in nearby theaters. The way we see and treat obesity is bound to change

**DOI:** 10.20945/2359-3997000000466

**Published:** 2022-04-28

**Authors:** Rogério Friedman

**Affiliations:** 1 Universidade Federal do Rio Grande do Sul Departamento de Medicina Interna Porto Alegre RS Brasil Departamento de Medicina Interna, Universidade Federal do Rio Grande do Sul, Porto Alegre, RS, Brasil; 2 Hospital de Clínicas de Porto Alegre Serviço de Endocrinologia e Metabologia Porto Alegre RS Brasil Serviço de Endocrinologia e Metabologia, Hospital de Clínicas de Porto Alegre, Porto Alegre, RS, Brasil

“Pandemic”, “health crisis”, “global health threat”, “social and economic burden”. Obesity has been called by many different names. No matter what name you choose, obesity is still a growing issue, globally. Over the last five or six decades, the amount of individuals suffering from this condition has not ceased to increase.

Non-surgical treatment options are still scarce, and the outcomes are often disappointing. One of the main reasons for the frustrating panorama is our poor understanding of the underlying mechanisms of obesity. Only in the last 30 years or so science has begun to properly unravel the neuro-hormonal pathways that contribute to this disease. This knowledge is bringing new treatment options to the market, but the problem is still massive, not all patients have access to all therapeutic tools and the global prevalence remains growing.

Another reason for treatment options to be often disappointing is that we still consider obesity as a static condition. Patients are classified in “degrees”, based on their measured weight and height, and the derived body mass index (BMI). This is just like looking at a photograph. One can surely deduce a series of data from the static picture, but this implies that many subjects are put in the same category, not regarding the important data arising from their personal history and weight trajectory.

The individual history of obesity is, in fact, a film, rather than a photograph. And this movie must be watched, so the practitioner can have a fuller picture of the patient’s condition. This allows for a better understanding of the risk and a better assessment of the factors that impact the patient’s health status. And from this “cinematic” view, a more personalized and effective approach may become possible.

In this issue of *Archives of Endocrinology and Metabolism*, a distinguished group of Brazilian colleagues, representing both the Brazilian Endocrine Society (*Sociedade Brasileira de Endocrinologia e Metabologia*, SBEM) and the Brazilian Society for the Study of Obesity and Metabolic Syndrome (*Associação Brasileira para o Estudo da Obesidade e da Síndrome Metabólica*, ABESO) bring forward an innovative proposal of a classification of obesity based on the weight trajectory and the maximum weight achieved in life (MWAL) (1). The proposal is based on quality epidemiological and clinical data and seeks not to replace the traditional classification based on BMI, but to add to it, for individual weight changes over time to be taken into account when assessing a patient’s risk for untoward events and unfavorable outcomes.

We invite you to read the article carefully and try to use this new camera angle with your patients. Perhaps, the way you see those under your care will change and you will accumulate further ability to individualize your approach to this major health problem.

## References

[1] Halpern B, Mancini MC, de Melo ME, Lamounier RN, Moreira RO, Carra MK (2022). Proposal of an obesity classification based on weight history: an official document by the Brazilian Society of Endocrinology and Metabolism (SBEM) and the Brazilian Society for the Study of Obesity and Metabolic Syndrome (ABESO). Arch Endocrinol Metab.

